# Heterochromatic Gene Silencing by Activator Interference and a Transcription Elongation Barrier*[Fn FN2]

**DOI:** 10.1074/jbc.M113.460071

**Published:** 2013-08-12

**Authors:** Aaron Johnson, Ronghu Wu, Matthew Peetz, Steven P. Gygi, Danesh Moazed

**Affiliations:** From the ‡ Department of Cell Biology and; ¶ Howard Hughes Medical Institute, Harvard Medical School, Boston, Massachusetts 02115 and; § Department of Biochemistry and Molecular Genetics, University of Colorado School of Medicine, Aurora, Colorado 80045

**Keywords:** Chromatin, Chromatin Modification, Gene Silencing, Heterochromatin, Transcription, Sir Proteins

## Abstract

Heterochromatin silences transcription, contributing to development, differentiation, and genome stability in eukaryotic organisms. Budding yeast heterochromatic silencing is strictly dependent on the silent information regulator (SIR) complex composed of the Sir2 histone deacetylase and the chromatin-interacting proteins Sir3 and Sir4. We use reconstituted SIR heterochromatin to characterize the steps in transcription that are disrupted to achieve silencing. Transcriptional activator binding is permitted before and after heterochromatin assembly. A comprehensive proteomic approach identified heterochromatin-mediated disruption of activator interactions with coactivator complexes. We also find that if RNA polymerase II (Pol II) is allowed to initiate transcription, the SIR complex blocks elongation on chromatin while maintaining Pol II in a halted conformation. This Pol II elongation barrier functions for even one nucleosome, is more effective when assembled with multiple nucleosomes, and is sensitive to a histone mutation that is known to disrupt silencing. This dual mechanism of silencing suggests a conserved principle of heterochromatin in assembling a specific structure that targets multiple steps to achieve repression.

## Teaser

**Background:** Heterochromatic gene silencing inhibits transcription, but the mechanism of silencing is not currently understood.

**Results:** Reconstituted budding yeast heterochromatin disrupts transcriptional coactivator recruitment and RNA polymerase elongation.

**Conclusion:** Yeast silencing operates by multiple mechanisms to achieve stable repression.

**Significance:** Heterochromatic silencing mechanisms have many common aspects that may be conserved from yeast to human.

## Introduction

Heterochromatin is a conserved feature of eukaryotic chromosomes that serves to repress the transcription of certain genes and to confer genome stability within repetitive regions of the genome (1). Although many subtypes of heterochromatin exist, all share a number of hallmark features. In general, heterochromatin domains are formed by specific silencing factors that stably assemble with canonical nucleosomes composed of the four core histones, H2A/H2B/H3/H4. The most fundamental post-translational modification pattern present in all heterochromatin is deacetylation of the histones, whereas acetylation is known to disrupt the structure and function of heterochromatin (2, 3). Different mechanisms can direct heterochromatin-mediated transcriptional gene silencing, employing site-specific DNA binding proteins, chromatin-interacting proteins, as well as noncoding RNAs and RNA processing factors (1, 4). The requirement for specificity factors, along with histone modifications that inhibit heterochromatin assembly, restricts heterochromatin formation to specific regions of chromosomes.

Silencing factors typically act in larger complexes, often incorporating one or more proteins that have functions elsewhere in the cell. Budding yeast heterochromatin has been studied extensively by genetic and biochemical analyses that have identified the factors that are absolutely required for silencing (3, 5). These include three proteins that form the silent information regulator (SIR)^4^ complex. The SIR complex is composed of the Sir2 lysine deacetylase that is known to deacetylate histones and also non-histone targets. Sir2 is found in complex with Sir3 and Sir4, histone-binding proteins that are involved both in recruitment of the SIR complex to chromatin and serve as integral structural components of heterochromatin (6–10).

Although much is known about the way that the SIR complex assembles a heterochromatin domain, it has remained unclear what the actual mechanism of silencing is, even in this most basic and well studied silencing system. Two different proposals have been made to explain how budding yeast heterochromatin achieves transcriptional repression: either by exclusion of RNA polymerase II (Pol II) from the silenced gene through an unknown mechanism (11) or by somehow preventing polymerase elongation (12, 13). In this regard, gene silencing in facultative heterochromatin by the Polycomb group complexes has been proposed in multiple organisms to occur downstream of activator binding, most likely at the step of transcription initiation by Pol II (14–16). Binding of Polycomb complexes has been demonstrated *in vitro* to lead to compaction of nucleosome arrays and inhibition of transcription (16–18).

In this report, we explore the mechanism of heterochromatic gene silencing in budding yeast using a recently developed *in vitro* model for repression of activator-dependent transcription (3). We find that a transcriptional activator can bind readily within heterochromatin, but proteomic profiling of the interaction of nuclear factors with a heterochromatin domain demonstrates that the silent structure interferes with recruitment of coactivator complexes by the activator. This explains the low levels of stable RNA polymerase II within an *in vitro*-assembled heterochromatin domain. Yet, if Pol II is allowed to engage the chromatin in elongation mode, a heterochromatin barrier can readily stop the polymerase, maintaining it in a halted conformation.

## EXPERIMENTAL PROCEDURES

### 

#### 

##### Proteins and DNA Templates

All proteins were purified as described in Ref. 3. DNA templates used in activator binding and proteomic profiling experiments were made by PCR from the plasmid pUC18-G5cyc1^G−^ bearing five Gal4 binding sites upstream of a CYC1 promoter-driven G-less cassette with two predicted start sites producing transcripts of ∼250 and 277 nucleotides (see Refs. 19 and 20) for details on use of G-less templates). PCR-generated templates were produced with a biotinylated primer on the end furthest from the transcription cassette. The transcription cassette begins 582 bp from the non-biotinylated end. The size of PCR product used is indicated in the description of the experiment. DNA templates for transcription elongation assays were generated by PCR from the plasmid pAd-GR220 (21), digestion with XmaI, and ligation of a short double-strand segment made from two annealed oligonucleotides that generate a 3′ 20-nucleotide oligo-dC tail after ligation as described previously (22). Similar tailed templates were made by PCR primers and ligation that generated a 49-bp A-less template strand upstream of a 601-nucleosome positioning sequence (43). All templates were gel-purified after ligation, and ligation was confirmed by gel analysis.

##### Chromatin Reconstitution and Nucleosome Assembly

Long (more than four nucleosomes) nucleosome arrays were assembled enzymatically, and shorter nucleosome substrates were generated by salt dialysis as described previously (3). Conjugation of chromatin fragments to magnetic beads and acetylation with Piccolo acetyltransferase was also done as described previously (3).

##### Activator-dependent Pol II Transcription Assays

Assays were performed essentially as described previously (3, 23).

##### Heterochromatin in Vitro Immunoprecipitation

A 3.1-kb chromatinized fragment of pUC18-G5cyc1^G−^ (40 ng of DNA) was incubated with 25 ng of purified Gal4-VP16 activator for 30 min at room temperature. When present, SIR complex was prepared as a preincubated sample of Sir3 (1.7 pmol) and Sir2/4 subcomplex (470 fmol), incubated on ice. The 3.1-kb chromatin fragment was incubated with or without the SIR complex for 60 min at room temperature in 8.5 μl of 50 mm Hepes, pH 7.5, 100 mm potassium acetate, 1 mm magnesium acetate, 0.1 mg/ml BSA, 10% glycerol, 0.02% Nonidet P-40 (Nonidet P-40), 0.3 mm EGTA, 2.5 mm β-glycerophosphate, 0.1 mm PMSF, and 1 mm DTT, prior to incubation with 160 μg (4 μl) of nuclear extract (prepared as described previously (3)) for 30 min at room temperature with a shift to 25 mm Hepes, pH 7.5, 6 mm magnesium acetate, 2.5 mm EGTA, 95 mm potassium acetate, 20 mm ammonium sulfate, 2 mm DTT, 1.7 mm β-mercaptoethanol, 0.01% Nonidet P-40, 0.34 units/μl Protector RNase inhibitor (Roche Applied Science), 3.4 mm phosphocreatine, 0.034 units/μl creatine kinase; and then a 20-min incubation with NTPs at a final concentration of 500 μm ATP, GTP, and CTP; 10 μm UTP in 12.5 μl of total volume. Samples were shifted to ice, and then 450 μg Dynabeads-protein A (Invitrogen) were coupled to 540 ng of antibody (anti-FLAG M2 (Sigma) or 8WG16 (Abcam)) in 100 μl of 25 mm Hepes, pH 7.5, 60 mm potassium acetate, 7.5 mm magnesium acetate, 0.02% Nonidet P-40, 5% glycerol, 10 mm β-glycerophosphate (IP buffer) was added, and samples were incubated for 1 h at 4 °C with rotation. Beads were washed once with 200 μl of IP buffer and resuspended in 25 μl of SDS sample buffer. Samples were separated by 8% SDS-PAGE and transferred to nitrocellulose, and Western blot was performed with the antibodies described above or with Gal4 DBD antibody (Santa Cruz Biotechnology).

##### Chromatin Pulldown with Activator and SIR Complex

A 3.1-kb biotinylated, chromatinized PCR product from pUC18-G5cyc1^G−^ (67 ng of DNA), conjugated to magnetic beads, was incubated with 33 ng of Gal4-VP16 for 30 min (or Gal4-VP16 after SIR complex) in 50 mm Hepes, pH 7.5, 10 mm potassium acetate, 1 mm magnesium acetate, 0.1 mg/ml BSA, 10% glycerol, 0.02% Nonidet P-40, 0.3 mm EGTA, 2.5 mm β-glycerophosphate, 0.1 mm PMSF, and 1 mm DTT with rotation at room temperature. The SIR complex (2.3 pmol Sir3, 600 fmol Sir2/4) was added, and the sample was further incubated for 1 h in 12 μl of total volume. Sample was shifted to 25 mm Hepes, pH 7.5, 6 mm magnesium acetate, 2.5 mm EGTA, 95 mm potassium acetate, 20 mm ammonium sulfate, 2 mm DTT, 1.7 mm β-mercaptoethanol, 0.01% Nonidet P-40, 0.34 units/μl Protector RNase inhibitor, 3.4 mm phosphocreatine, 0.034 units/μl creatine kinase, and Gal4-VP16 was added, if not in the first step. Samples were incubated 25 min at room temperature, and then beads were washed in IP buffer once and analyzed as above by Western analysis.

##### Quantitative Mass Spectrometry Profiling of Chromatin Domains

Yeast cultures, strain SF10 (7), were grown in synthetic medium with either 32 mg/liter ^12^C_6_^14^N_2_ lysine-HCl (Sigma) or ^13^C_6_^15^N_2_ lysine-HCl (Cambridge Isotope Laboratories) in 3% glucose to *A*_600_ of 2.0. Extracts were prepared from cell pellets as described previously (20).

Bead-conjugated 3.1-kb pUC18-G5cyc1^G−^ chromatin template (1.5 μg of DNA was incubated with the SIR complex (13.6 pmol Sir2/4, 52 pmol Sir3)) for 1 h with rotation in 400 μl of 50 mm Hepes, pH 7.5, 10 mm magnesium acetate, 5 mm EGTA, 0.1 mm EDTA, 0.02% Nonidet P-40, 5% glycerol, 1 mm DTT, 1 mm PMSF, 1 μg/ml bestatin/leupeptin/pepstatin, and 1 mm benzamidine. Subsequently, 150 ng of Gal4-VP16, an ATP regeneration system (30 mm creatine phosphate, 3 mm ATP, 4.1 mm magnesium acetate, and 6.4 μg/ml creatine kinase, final concentration), and 1.5 mg of light or heavy yeast extract were added to 500 μl and incubated for 1 h at room temperature and 1 h at 4 °C with rotation. Where indicated, chromatin was acetylated as described previously (3) prior to incubation with SIR complex and activator as described above. Beads were then washed one time with 1.5 ml of cold IP buffer. Beads were then stripped of protein with a solution of 50 mm Hepes, pH 7.5, and 2 m NaCl. Samples were diluted to 400 mm NaCl in 50 mm Hepes, pH 7.5, and light and heavy samples were combined and precipitated with trichloroacetic acid.

TCA pellets were resuspended and digested with Lys-C protease. The resulting peptides were purified using tC18 SepPak cartridge (Waters, Milford, MA). The dried peptides were resuspended in the solvent of 5% acetonitrile and 4% formic acid and were loaded onto a microcapillary column packed with C18 beads (Magic C18AQ, 5 μm, 200 Å) using a Famos autosampler (LC Packings, San Francisco, CA). The samples were separated by on-line reversed phase chromatography using an Agilent 1100 binary pump with a 70-min gradient of 5–30% acetonitrile (in 0.125% formic acid) and detected in a hybrid quadrupole linear ion trap-Orbitrap mass spectrometer (LTQ Orbitrap XL, Thermo Fisher Scientific).

All recorded MS/MS spectra were searched using the Sequest algorithm (version 28) (24). Spectra were matched against a database encompassing sequences of all proteins in the yeast ORF database downloaded from the *Saccharomyces* Genome website. Each protein sequence was listed in both forward and reversed orientations to facilitate estimation of peptide and protein identification false discovery rates. The following parameters were adopted: precursor mass tolerance, 10 ppm; product ion mass tolerance, 1.0 Da; up to two missed cleavages; variable modifications: oxidation of methionine (15.9949) and carbamidomethylation of cysteine (57.0214). The target decoy method was employed to distinguish correct and incorrect identifications and thus control peptide and protein level false discovery rates (25). The final list of peptides and proteins were selected by linear discriminant analysis in which numerous parameters, such as *X*_corr_, ΔCn, precursor mass error, and charge state, were considered (26). A 1% false discovery rate was strictly controlled at the protein level. Western blot confirmation was performed with antibodies for TATA-binding protein (TBP) (Abcam), yeast Sua7 and Taf11 (Thermo Fisher Scientific), and Spt3 (Santa Cruz Biotechnology).

##### Transcription Elongation Assays

Poly-dC tailed DNAs derived from pAd-GR220 (3.1-kb template) were assembled into nucleosomes by enzymatic means and 1–3 nucleosome-sized 601-containing templates were assembled by salt dialysis as described previously (3). Tailed nucleosomal template (25–50 ng of DNA) was incubated with 60 fmol of RSC complex (RSC2-TAP) for 30 min. at 30 °C; followed by incubation with 230 ng of purified core Pol II (Rpb9-TAP), and 60 μm ATP/GTP, 2.3 μm CTP, 25 μCi α-^32^P-CTP for 1 h at 30 °C; then 1-h incubation with SIR complex (470 fmol Sir 2/4, 1.7 pmol Sir3); and finally chased with 125 μm CTP, 1 μm UTP for 1 h (3.1-kb template) or 5 min (601-containing templates). Final reaction conditions were 50 mm Hepes, pH 8.0, 12 mm magnesium acetate, 500 μm ATP, 1 mm EDTA, 7.5% glycerol, 0.5 mg/ml BSA, 0.13 units/μl protector RNase inhibitor (Roche Applied Science) in 30 μl. When present, 15 μl of 2.7 m KCl was added to reactions either instead of or immediately after the 125 μm CTP/1 μm UTP chase, reactions were incubated for 5 min at room temperature, 1 μl of 22.5 mm NTPs/90 mm magnesium acetate was added, and reactions were chased for 1 h at room temperature. Reactions were quenched with 90 μl of 10 mm Tris-HCl, pH 7.5, 200 mm NaCl, 5 mm EDTA, proteinase K-treated, phenol/chloroform-extracted, ethanol-precipitated, and separated on a 6 or 8% polyacrylamide urea/TBE gel. Gels were exposed to a storage phosphor screen and visualized and quantified by Quantity One (Bio-Rad) or ImageQuant (GE Healthcare) software.

## RESULTS

### 

#### 

##### Activator Binding Is Not Affected by Assembly of the SIR Complex on Chromatin

**FIGURE 1.**
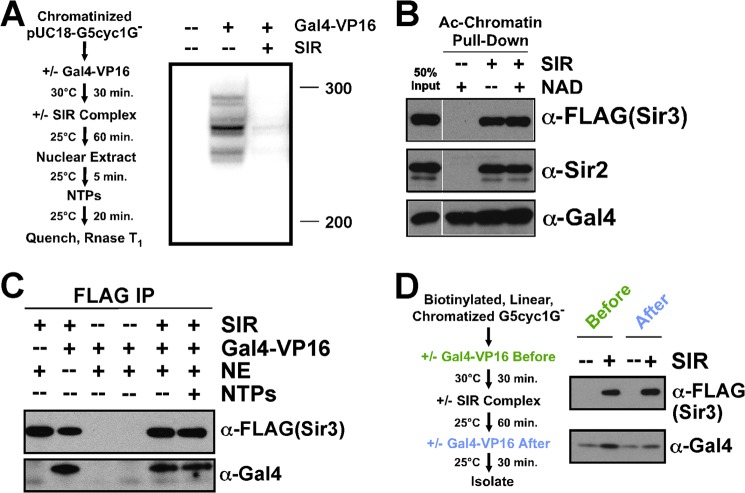
**Heterochromatin allows activator binding.** *A*, the system for observing repression of activator-dependent transcription provided by a nuclear extract. Chromatinized, circular pUC18-G5cyc1^G−^ is used. *B*, linear, biotinylated PCR product containing the entire pUC18-G5cyc1^G−^ sequence was chromatinized and conjugated to streptavidin-coated magnetic beads, acetylated enzymatically (*Ac-Chromatin*), bound by activator, then SIR complex in the absence or presence of NAD^+^. Stable chromatin-bound proteins were detected by Western analysis. *C*, the Gal4-VP16 activator was incubated with the linear chromatin template in *B* in solution prior to addition of the SIR complex, nuclear extract, and NTPs, following the scheme shown in *A*. SIR-bound chromatin was immunoprecipitated with FLAG antibody recognizing Sir3-FLAG, and Sir3 and Gal4 were detected by Western blot. *D*, streptavidin bead-conjugated template from 1B was used, and orders of addition of SIR complex and Gal4 were tested as indicated in the scheme on the *left*.

Reconstitution of a minimal system that recapitulates heterochromatic gene silencing (3) led us to ask at what step in the mechanism of RNA polymerase II transcription does the SIR complex act to repress activator-dependent transcription. We first asked whether binding of the activator, the first step in the mechanism, was affected. We used a fusion of the yeast Gal4 DNA binding domain to the activator region of the VP16 viral polypeptide (Gal4-VP16) as the activator in our system (3, 19, 20). A chromatinized plasmid template bearing an array of Gal4 binding sites upstream of a transcription cassette was incubated with the remaining components of the transcription system as well as the SIR complex, which causes transcriptional repression (Fig. 1*A*). Using a linear, biotinylated version of this template conjugated to magnetic beads demonstrated that binding of the SIR complex to chromatin did not affect the association of the activator (Fig. 1*B*). The SIR-bound chromatin template was immunoprecipitated with an antibody against the epitope tag on Sir3. The activator remained bound to the SIR-coated chromatin under all conditions (Fig. 1*C*). Activator association with heterochromatin was observed irrespective of order of addition (Fig. 1*D*). In fact, it appeared that the SIR complex was able to enhance stable activator binding, perhaps by decreasing the off rate of the activator. This result suggested that the SIR complex may physically affect the activator that is bound within heterochromatin.

##### A Comprehensive Method to Profile Reconstituted Heterochromatin Domains

There are many steps and multiprotein complexes required to initiate transcription on a naked DNA template, and additional chromatin-interacting complexes required for efficient initiation on chromatin. The sheer number of factors involved poses a significant challenge to studying the potential changes in chromatin interactions that occur when heterochromatin forms. The traditional methods of Western blotting and chromatin immunoprecipitation rely on antibody recognition, either via native epitopes or tags that prevent a comprehensive analysis in a single experiment. To circumvent these challenges, we developed a method whereby we can comparatively quantify factors bound to two different chromatin samples: free chromatin (euchromatin-like) and heterochromatin. This method relies on differential labeling of samples produced by a preassembled chromatin domain and factors that interact with this domain in a cell extract. We generated transcription-competent extracts (20) from yeast grown in normal synthetic medium or medium prepared with isotopically enriched lysine. The extracts contained either light or heavy proteins (supplemental data set S1) that were incubated with a biotinylated chromatin domain. Samples were washed, and proteins were stripped from the conjugated DNA, mixed together, and prepared for mass spectrometric analysis of enzymatically digested peptides.

**FIGURE 2.**
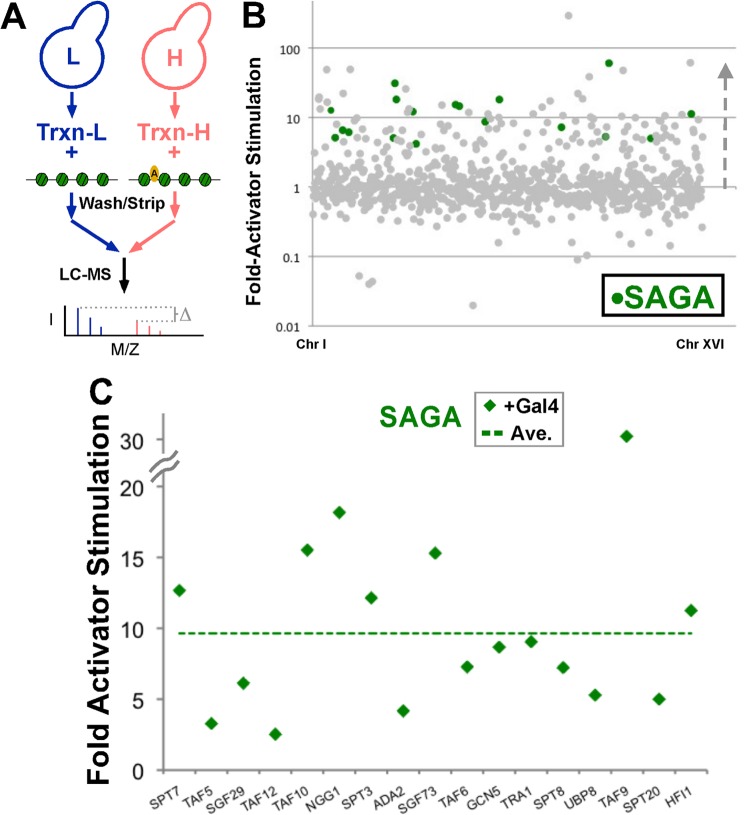
**Activation of transcription analyzed by comprehensive chromatin profiling.** *A*, depiction of the comprehensive comparative mass spectrometry experiment using isotopically labeled amino acids to generate transcription-competent yeast extracts with light (*L*) or heavy (*H*) proteins. Extracts were incubated with free or activator-bound linear, biotinylated pUC18-G5cyc1^G−^ conjugated to streptavidin beads, and stably interacting proteins were pooled and prepared for comparative quantitative mass spectrometry. *B*, log-scale plot of all factors detected in the experiment and their fold-change due to presence of activator. SAGA complex components are highlighted in *green. C*, linear plot of SAGA complex members identified from *B*. Values from individual subunits shared by other complexes were divided and distributed equally to each complex. The average (*Ave.*) value of the complex was calculated using unique subunits only. Chd1 subunit of SAGA was omitted from analysis due to its known ability to act independently of SAGA.

To first test the efficacy of this comparative mass spectrometry profiling system, we profiled the effect of activator association with the chromatin template. A number of factors are known to be recruited by transcriptional activators such as the SAGA coactivator, mediator complex, TFIID, and multiple chromatin remodeling complexes (27). A reconstituted chromatin domain was prepared and conjugated to a magnetic bead as described (3). The Gal4-VP16 activator was preincubated with half of the chromatin sample, and then both chromatin templates were incubated in transcription extract from either light or heavy yeast cultures. Stably associated proteins from both samples were isolated, combined in equal volume of elution from chromatin, and peptides were profiled by mass spectrometry (Fig. 2*A*). Approximately twice as many factors from the extract were at least 2-fold stimulated in their association with the activator-bound chromatin template as were down-regulated (Fig. 2*B* and supplemental data set S2). 75 of the 145 nuclear factors up-regulated are subunits of protein complexes directly involved in activation of transcription such as SAGA, Mediator, TFIID, SWI/SNF, RSC, and NuA4. A similar pattern was observed by a different proteomic technique used recently to profile activator affects on chromatin (28).

##### Activator Interactions Are Disrupted by a Specific Heterochromatin Structure

**FIGURE 3.**
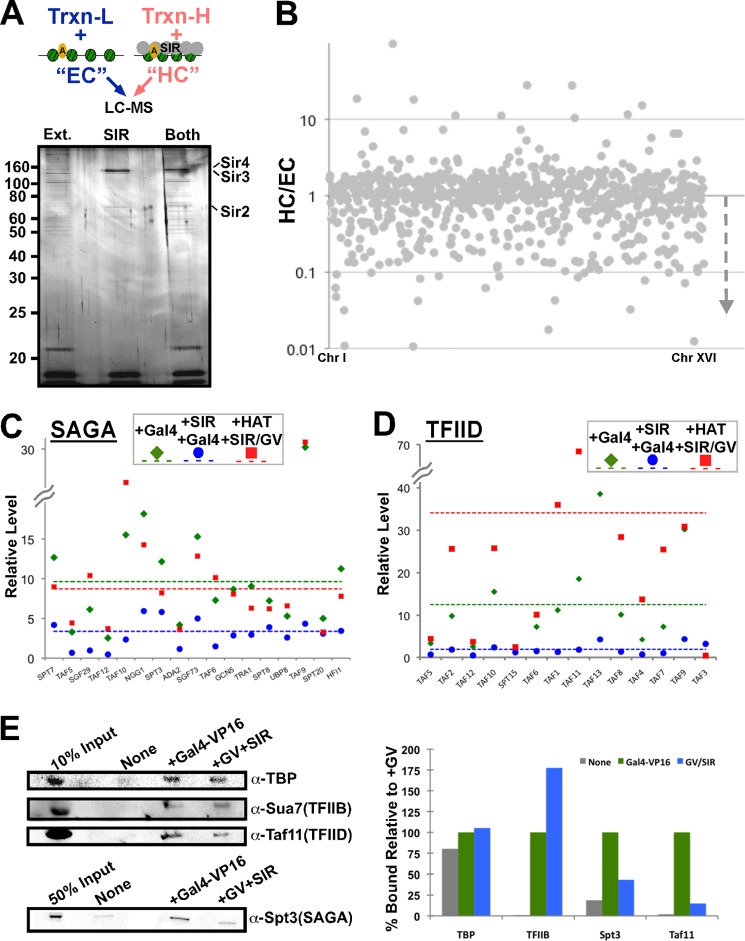
**Proteomic analysis of heterochromatin assembly effects on chromatin association.** *A*, *top*: similar scheme as in Fig. 2*A* using light and heavy extracts (*Ext.*) to profile the changes in a euchromatin-like (*EC*), activator-bound chromatin domain compared with an activator-bound heterochromatin (*HC*) domain. *Bottom*: 1% of the samples subjected to mass spectrometry (chromatin incubated with extract alone, SIR alone, or both) were separated on a 12% SDS-polyacrylamide gel and were subsequently silver stained. *B*, log-scale profile of all factors identified in the experiment depicted in *A. C*, linear plot of SAGA components identified in the heterochromatin experiment (*blue*) as well as an experiment profiling the difference when the chromatin template was acetylated prior to SIR complex binding and maintained in the absence of NAD (*red*). All values are calculated relative to the basal binding in the absence of Gal4-VP16. Averages of the levels in each experiment, including that from Fig. 2*C*, are shown as *dashed lines*. Average value for the complex was calculated using unique subunits only. *D*, similar profile to Fig. 2*C* for the components of TFIID that were identified. Spt15(TBP) was omitted from averaging due to its independent binding activity. Taf3 was determined to be an outlier and omitted. Taf14 was omitted from the analysis due to the large number of complexes with which associates. Average value of the complex was calculated using unique subunits only. *E*, a biotinylated trinucleosome PCR template from pUC18-G5cyc^G−^ was assembled into chromatin, conjugated to magnetic beads and an equivalent experiment to that performed for the mass spectrometry profiling was performed to assess the binding of TBP, Sua7(TFIIB), Taf11(TFIID), and Spt3(SAGA) from yeast extract to the chromatin alone, and with additions of Gal4-VP16 (GV) alone or with SIR complex(GV+SIR). Western analysis was performed with chemiluminescence (*left*) and quantified with Bio-Rad Image Lab (*right*).

The effectiveness of the quantitative comparative mass spectrometry analysis system to study changes to chromatin interactions was evident using the presence of activator on chromatin as a positive control (supplemental data set S2). We next wished to determine the effect on the protein interaction network when activator-bound chromatin was assembled into a heterochromatin structure. Conjugated chromatin was split into two, and one sample was assembled into heterochromatin, and the other was assembled as a mock assembly. These chromatin templates were then incubated with activator and transcription extract from either heavy or light extracts. Stably associating factors were isolated from each of the samples and combined and analyzed as above by mass spectrometry (Fig. 3*A*).

In contrast to the activator experiment where significantly more factors were enriched on the activator-bound chromatin, heterochromatin assembly caused a pattern where more factors were depleted from associating with chromatin, compared with the free chromatin (euchromatin-like) sample (Figs. 3*B* and supplemental data set S3). The majority of the nuclear factors that were disrupted from association with chromatin by the silent structure were the same factors that were recruited by activator. Particularly apparent was the pattern of nearly all of the SAGA (Fig. 3*C*) and TFIID (Fig. 3*D*) components, which were inhibited by heterochromatin as much as 10-fold and on average ∼3-fold (SAGA) or 6-fold (TFIID) from association with the underlying chromatin domain. This pattern was reproducible in two independent experiments, the second of which swapped extracts for each sample, for the majority of identified subunits of SAGA, TFIID, SWI/SNF, RSC, and NuA4 (Table 1 and supplemental Table S1). The pattern was also confirmed by Western blot for unique subunits of SAGA and TFIID (Fig. 3*E*). Mediator was only identified in one out of two experiments, perhaps due to the lack of direct contact between this complex and chromatin, but a similar pattern to other coactivator components was observed (supplemental Table S1).

**TABLE 1:** **Average values for coactivator complexes from mass spectrometry experiments** Unique subunits were averaged to generate values reflective of the pattern for each coactivator complex listed. Notable unique subunit omissions from averaging were as follows: SPT15, CHD1, TAF3, and RSC1. TBP(Spt15) protein levels alone are also listed.

Complex	GV/No GV	GV-HC/GV	HAT-GV-HC/GV
TFIID	12.53	0.13	2.73
SWI/SNF	6.21	0.20	3.47
NuA4	7.73	0.23	1.24
RSC	6.67	0.24	4.26
MED	4.29	0.33	3.70
SAGA	9.64	0.38	0.90
INO80	1.88	0.46	1.56
TBP (alone)	2.05	0.58	1.18
SWR1	1.92	0.99	2.62

Although the majority of TFIID components were significantly disrupted in association with chromatin by the silent structure, the most notable exception was TBP (also known as Spt15). TBP interaction with chromatin was affected less than the threshold (2-fold) by the assembly of heterochromatin (Fig. 3, *D* and *E*), compared with the highest effect, ∼10-fold less bound for TAF11 (Fig. 3*D*). The TBP-interacting general transcription factor, TFIIB, was not identified in the proteomic analysis but was investigated by Western analysis. Interestingly, TFIIB also was not disrupted by SIR heterochromatin (Fig. 3*E*); in fact, it was somewhat enhanced, similar to the enhancement observed for Gal4-VP16. The presence of activator, TBP, and TFIIB at similar levels on euchromatic and heterochromatic templates suggests that there is not a general exclusion principle governing gene silencing. In fact, recruitment of other factors by the activator is the step that is most interfered with by silent chromatin formation.

To test whether this activator interference mechanism was specific to heterochromatin structure, we treated the chromatin template with histone acetyltransferase prior to incubation with the silencing complex. Previously, we demonstrated that the SIR complex can bind to chromatin that is acetylated, but in the absence of the NAD cofactor that is required for Sir2 activity, the acetyl-lysines remain and the SIR complex cannot engage the template in a productive mode for transcriptional silencing (3). This is due to the lack of direct interactions between the Sir3 subunit and the amino terminus of histone H4, which prevents a distinct structural change in the SIR-bound chromatin assembly. SIR-bound, acetylated chromatin was incubated with activator and transcription extract, and the protein interaction profile was compared with that of the original activator-bound “euchromatin” state. A dramatic change in the chromatin protein interactome was observed with the acetylated SIR-bound chromatin sample, compared with the deacetylated SIR-bound chromatin. The majority of SAGA and TFIID subunits that were disrupted in their chromatin association by functional heterochromatin bound to the template that was acetylated, even though the SIR complex remained associated (Fig. 3, *C* and *D* and Table 1; supplemental data set S4). This suggests that a SIR-bound chromatin domain must be in a conformation that is competent for transcriptional repression (unacetylated) to prevent recruitment of factors by the transcriptional activator.

##### RNA Polymerase II Does Not Efficiently Associate with SIR Heterochromatin

**FIGURE 4.**
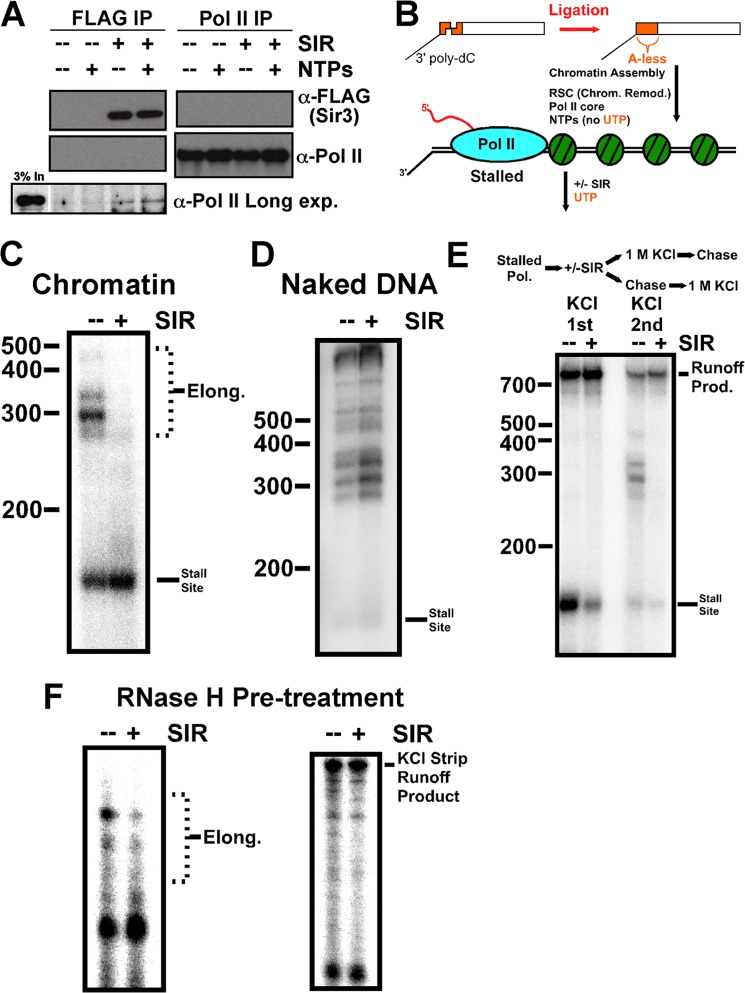
**SIR-mediated halting of Pol II elongation.** *A*, co-immunoprecipitation experiment similar to Fig. 1*C* with antibodies against FLAG or the large subunit of RNA polymerase II. Nuclear extract and chromatin template were present in all samples. *Left*, *bottom panel*, Long exposure (*Long exp.*) for Pol II blot. *B*, scheme for generating a tailed nucleosome array of 3.1 kb capable of transcription elongation by purified core Pol II. *Chrom. Remod.*, chromatin remodeling. *C* and *D*, effect of SIR complex assembly on chromatin or naked DNA ahead of elongating (*Elong.*) Pol II. *E*, RNA polymerase was allowed to complete transcription via high salt treatment in the presence or absence of a heterochromatin barrier assembled ahead of transcription progress. High salt and high-salt chase was added either before or after transcription and was allowed to proceed past the stall site by addition of a nucleotide chase containing UTP. *F*, experiments from Fig. 1, *C* and *E*, were repeated in the constant presence of RNase H to reduce the presence of RNA-DNA hybrids. *Prod.*, product.

In the course of profiling interactions with euchromatin and heterochromatin by mass spectrometry, certain subunits of RNA polymerase II were identified in the euchromatin sample but were prevented from interaction in the heterochromatin sample. The number of subunits of Pol II that were identified was not extensive, compared with the complexes upstream in transcription activation (Fig. 3 and supplemental data set S3). To pursue this observation, immunoprecipitation of either Sir3 or the large subunit of Pol II was performed using the transcription extract. Each immunoprecipitation failed to co-purify the other protein in substantial amounts (Fig. 4*A*), implying that preformed heterochromatin does not allow efficient initiation and stable association of Pol II. Upon long exposure, a faint band corresponding to the Pol II large subunit was detected in the Sir3 immunoprecipitation when the SIR complex was present. This small amount of polymerase was stable even when NTPs were added, indicating that it was not able to transcribe the entire template and dissociate from the end. This hinted at the possibility that the SIR complex may be able to affect Pol II elongation, in addition to association with chromatin.

##### The SIR Complex Can Prevent Pol II Elongation

If it is possible for Pol II to initiate near or within heterochromatin, what are the consequences with regard to transcription? Specifically, does a heterochromatin structure ahead of Pol II present a barrier to transcription elongation? To test this, we utilized a transcription system whereby the steps in initiation are bypassed. A DNA template was created with a 3′ poly-dC single-stranded tail on one end of a double-stranded template (Fig. 4*B*) (21). This tail allows purified core RNA polymerase II to load and begin transcription elongation once it encounters the double-stranded DNA. A stretch of ∼120 bases of the template lack As which allows a stalled polymerase to assemble at the end of that stretch in the presence of all ribonucleotides except UTP. This DNA template was chromatinized and incubated with the chromatin remodeling complex RSC, which promotes nucleosomal transcription elongation (22) and then purified core Pol II and the three NTPs; finally, UTP was added to allow transcription elongation to resume. When the SIR complex was assembled onto the chromatin template after Pol II preincubation, the elongation products were greatly reduced (Fig. 4*C*), indicating that the SIR complex can interfere with transcription elongation. This was a chromatin-mediated effect, as demonstrated by the fact that naked DNA was not a suitable substrate for the SIR complex to prevent transcription elongation (Fig. 4*D*). The slight increase in the presence of the SIR complex observed with naked DNA (∼16% increase assessed by quantification of the lanes) is comparable with that of an equal amount of BSA, by weight, added to the reaction (∼19%) (data not shown).

##### Pol II Is Stably Halted by a Heterochromatin Barrier

We next wished to determine what the fate of Pol II was when the SIR-chromatin structure disrupted transcription elongation. We tested whether Pol II remained associated with chromatin when confronted with a heterochromatin barrier or whether it was dislodged. Under conditions where the heterochromatin structure interfered with transcription elongation, we then stripped all nucleosome and SIR complex from DNA with high salt (Fig. 4*E*). With this treatment, elongating Pol II remains tightly bound to the DNA template and is competent for elongation (29). The reaction was then chased with NTPs to allow all Pol II remaining on DNA to complete transcription to generate a runoff product. We observed approximately equal runoff product formation in the absence or presence of the heterochromatin barrier ahead of Pol II (Fig. 4*E*, +SIR relative to −SIR: ∼115% for *KCl 1st*, 111% for *KCl 2nd*), indicating that disruption of transcription elongation by the SIR complex halts RNA polymerase, but Pol II remains stably bound to chromatin, presumably at the boundary of the heterochromatin domain. Pol II was equally stable upstream of a heterochromatin barrier whether stalled by lack of UTP (*KCl 1st*) or whether the heterochromatin itself was the cause of halting (*KCl 2nd*). Interestingly, in the case where UTP was lacking (*KCl 1st*), more products accumulated at the stall site without SIR, suggesting that polymerase was more likely to fall off at the stall site in the absence of heterochromatin. This suggests that the heterochromatin barrier may stabilize Pol II on chromatin. The experiments of Fig. 4, *C* and *E*, were repeated in the presence of RNase H, which reduces the occurrence of RNA-DNA hybrids generated by Pol II, and a similar SIR elongation block was observed (Fig. 4*F*).

##### A Nucleosomal Silencing Barrier Stops Pol II Elongation

**FIGURE 5.**
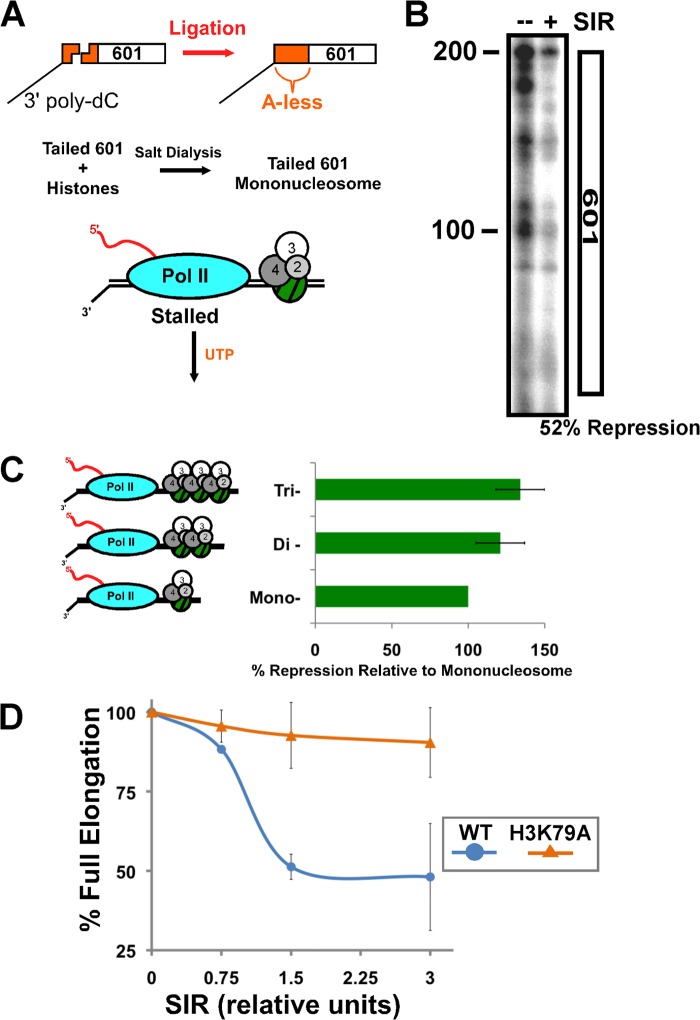
**A nucleosomal heterochromatin barrier blocks transcription elongation.** *A*, scheme for 601-containing nucleosome elongation substrate generation. *B*, Pol II elongation from a mononucleosome substrate as shown in *A* in the absence and presence of SIR complex. *C*, transcription through nucleosomal substrates of increasing size (*left*), beginning with the 601 substrate in *B*. Nucleosomal elongation products were quantified (*right*). Averages of triplicate experiments and S.D. are shown. Experiments in *B* and *C* were performed in the presence of RNase H. *D*, 601-containing mononucleosomes (50 ng of DNA) containing wild-type histones or histone H3 lysine 79 to alanine mutation (H3K79A) were used in an elongation assay as shown in *B* with SIR complex titrated to a final amount of 470 fmol Sir2/4, 1.7 pmol Sir3. The average of two experiments and deviation from the mean is shown. RNase H was not present in the experiment.

To determine the minimal unit of SIR-chromatin that can facilitate Pol II halting, we used a single nucleosome with a poly-dC tail as an elongation-competent substrate for Pol II (Fig. 5*A*). We preassembled stalled Pol II on the template in front of a positioned nucleosome, allowed the SIR complex to bind to that nucleosome, and finally added UTP to promote elongation. We found that even a mononucleosome-SIR complex was capable of interfering with full Pol II elongation through the nucleosomal DNA (Fig. 5*B*).

Comparison between the mononucleosome halting pattern and longer nucleosome arrays demonstrated that the SIR complex was able to more efficiently prevent significant elongation with increasing number of adjacent nucleosomes (Fig. 5*C*), suggesting that a more complex heterochromatin structure formed by incorporation of multiple nucleosomes is more efficient at preventing Pol II elongation.

We next tested whether specific interactions that are known to promote silencing *in vivo* were important for stopping Pol II elongation through a mononucleosome. Mutation of histone H4 lysine 16 modestly prevented the SIR complex in halting elongation (data not shown). More striking was the effect of histone H3 lysine 79 (H3K79) mutation, which significantly disrupted the activity of the SIR complex to stop Pol II (Fig. 5*D*).

## DISCUSSION

**FIGURE 6.**
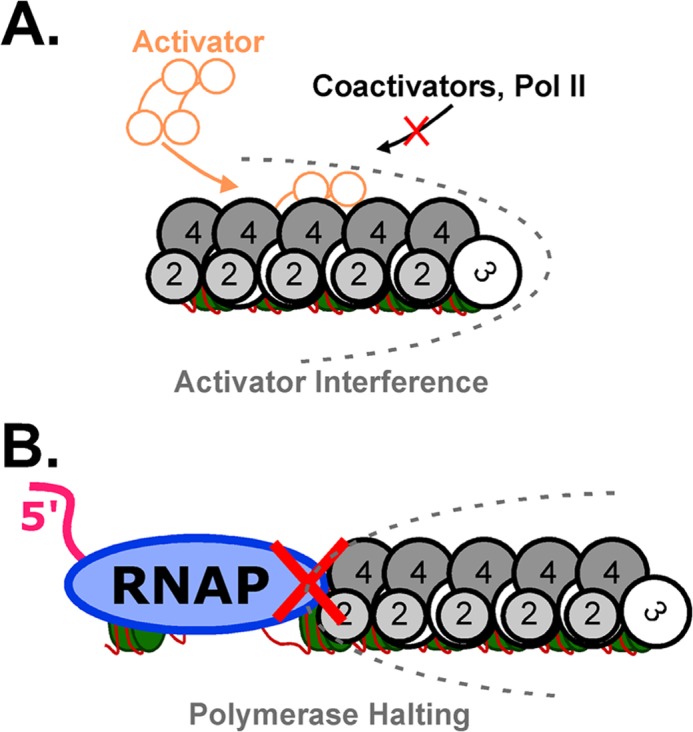
**Model for dual modes of heterochromatic gene silencing.** *A*, SIR-mediated heterochromatin allows transcriptional activator to bind, but the heterochromatin structure causes interference with the ability of the activator to recruit coactivators such as SAGA as well as the transcription machinery. *B*, an elongating RNA polymerase (*RNAP*) II can be halted by SIR-mediated heterochromatin.

Heterochromatic domains range in complexity, depending on the organism and the specific locus (1). Many organisms employ multiple pathways to achieve heterochromatic transcriptional silencing, including histone-binding proteins, RNAi components, and RNA degradation pathways (30, 31). We demonstrate in this report that budding yeast heterochromatin, thought to be the simplest form, silences gene expression using multiple mechanisms (Fig. 6).

### 

#### 

##### Activator Is Accommodated within Heterochromatin but Cannot Recruit Coactivator Complexes

The most consistent observation among the different studies of the mechanism of yeast heterochromatic gene silencing is that of transcriptional activator association within heterochromatin (12, 13, 32, 33), and this is true in the case of Polycomb silencing in multicellular eukaryotes (15, 16, 34). This observation has argued against a strict mechanism of silencing where all factors are excluded from chromatin association by assembly of the heterochromatin structure. Further confirmation of the somewhat permissive nature of heterochromatin comes from our observation of the consistent presence of the TATA-binding protein within heterochromatin (Fig. 3, *D* and *E*, and Table 1). Notably, both transcriptional activators and TBP can directly associate with DNA and are thought to be able to be recruited independently of their binding partners. Both factors are also relatively small in size, which, in combination with direct and independent binding to DNA, may be the reason that they can bind stably within heterochromatin. Persistent TBP association was also recently observed for polycomb repressive complex 1-reconstituted chromatin (34). The activity of the Rad51 recombination factor within heterochromatin can be facilitated by SWI/SNF activity (35). Coordinating specific chromatin remodeling activity may be a way for other factors to access heterochromatin, although recruitment of chromatin remodelers by transcriptional activators is disrupted by SIR-mediated heterochromatin (see below).

We find that though activator itself binds within heterochromatin, the coactivator recruitment that is required for activator function is significantly disrupted by assembly of the silent structure (Fig. 6*A*). This mechanism affects the recruitment of many coactivator complexes, which are central players in activating transcription (27). Although TBP contacts to TFIID may be disrupted by heterochromatin formation, TBP interference is not a general mechanism for transcriptional repression, as TFIIB binding to TBP is not disrupted by heterochromatin formation (Fig. 3*E*). The question remains whether activator interference occurs due to masking of the activation domain or prevention of other contacts with chromatin that would stabilize these complexes. Interestingly, a mechanism for corepressor function has recently been described that bears a similarity to heterochromatin-mediated activator interference (36). In this case, the corepressor binds directly to the activation domain and occludes the binding sites for coactivators. No interaction between the Sir proteins and activation domains are known, but this would be a potential explanation for the ability to inhibit coactivator recruitment. An alternative, although not mutually exclusive, possibility is that heterochromatin interference with coactivator recruitment relies on preventing these complexes from modifying and interacting with histones to stabilize their association. Finally, it is possible that heterochromatin interference acts to sterically hinder larger coactivator complexes from association with the activator and/or histones.

The SIR complex cannot disrupt coactivator recruitment when the chromatin remains acetylated, which suggests a distinct structural requirement for activator interference. Deacetylation of chromatin is a requirement to observe transcriptional repression in the *in vitro* silencing system (3). In addition, very little halted Pol II is detected when reconstituted heterochromatin is immunoprecipitated from a transcription system (Fig. 4*A*). These results suggest that the primary mechanism of transcriptional repression of a preformed heterochromatin domain is activator interference.

##### RNA Polymerase II Elongation Is Halted by a Heterochromatin Barrier

Although activator interference is likely the primary mechanism for a fully assembled heterochromatin domain to repress transcription initiating from within, we have also demonstrated that elongating Pol II, when it encounters a heterochromatin barrier, is halted in a stable conformation on chromatin (Fig. 6*B*). Elongation halting can work at the mononucleosome level, suggesting the interaction of the SIR complex with a single nucleosome is a fundamental unit of heterochromatic silencing. What then prevents the SIR complex from inappropriately halting transcription elongation in euchromatic regions of the genome? Two mechanisms can prevent spurious polymerase halting. First, the SIR complex is preferentially recruited to regions of the genome that contain a multi-site silencer element, bound by proteins that directly recruit the SIR complex (5, 37). In the absence of recruitment, SIR complex association with scattered euchromatic nucleosomes may occur with low efficiency. Second, anti-silencing chromatin modifications are likely to prevent spurious SIR-mediated silencing *in vivo*. Virtually all nucleosomes outside of heterochromatin bear the post-translational modification of histone H3 lysine 79 methylation (38). This modification has recently been shown to interfere with SIR-mediated silencing but not SIR complex binding (33). The SIR complex can bind to chromatin without silencer recruitment (3, 39) (this study), and we have demonstrated that H3K79 mutation to alanine disrupts the SIR complex from halting Pol II (Fig. 5*B*). Interestingly, Sir3 binds H3K79A and wild-type mononucleosome with near-identical affinity (3). Together, these studies suggest that the methylation of H3K79 plays a key role in regulating the silencing activity of the SIR complex at a step downstream of its binding to chromatin. Clues into the mechanism of this regulation come from recent structural studies of the Sir3 BAH domain bound to the nucleosome. The crystal structure of the Sir3 BAH domain bound to a mononucleosome highlights specific contacts between the BAH domain and H3K79, which would be disrupted when this site is methylated (40). In addition, the BAH domain makes extensive contacts with the amino terminus of histone H4 involving bonding interactions with H4K16 and H4H18, which help stabilize Sir3 on the nucleosome. Moreover, binding of the BAH domain to the nucleosome induces contacts between histone H4 arginines 17 and 19 (H4R17 and -19) and nucleosomal DNA, which have been proposed to act as a clamp that creates a silenced nucleosome (41). The formation of such an arginine clamp may require stable contacts between the BAH domain and both the H3K79 and H4K16 regions in the nucleosome. Our findings suggest that the interaction of Sir3 with H3K79 is critical for the ability of the SIR complex to halt transcription elongation. We therefore propose that H3K79 methylation may serve to prevent inappropriate SIR-mediated elongation arrest and silencing by preventing the formation of a stable arginine clamp even when the SIR complex is bound to chromatin. In this case, when Pol II elongation prevails, the SIR complex may be displaced to allow it a chance to be targeted properly.

##### Implications of Pol II Halting by Yeast Heterochromatin

Heterochromatic silencing from budding yeast to humans shares the feature of a multifaceted mechanism of transcriptional repression. Prevention of Pol II transcription initiation appears to be a dominant mechanism for stable, long term silencing. SIR-mediated Pol II halting may serve mainly to prevent invasion of transcription into regions that are tightly repressed, or it may be utilized during *de novo* establishment of a heterochromatin domain. Our observation that RNA polymerase II can be halted in an elongation state by a yeast heterochromatin domain is reminiscent of poised Pol II at many tightly regulated metazoan developmental genes that bear both active and repressive histone modifications (42). Poised Pol II in “bivalent” chromatin is regulated by silencing factors such as the Polycomb complexes, as well as other transcriptional regulators. The Pol II halting mechanism in budding yeast may operate in a similar manner to the bivalent domains of multicellular organisms that coordinate complex gene expression programs to drive cell differentiation along a specific pathway.

## Footnotes

4 The abbreviations used are:

SIR: silent information regulator
Pol II: RNA polymerase II
TBP: TATA-binding protein
SAGA: Spt-Ada-Gcn5-acetyltransferase
TFIID: transcription factor IID.
